# Barriers to Implementing Effective Healthcare Practices for the Aging Population: Approaches to Identification and Management

**DOI:** 10.7759/cureus.79590

**Published:** 2025-02-24

**Authors:** Nikolaos Theodorakis, Zoi Kollia, Michalitsa Christodoulou, Ioanna Nella, Aggeliki Spathara, Sofia Athinaou, Gesthimani Triantafylli, Christos Hitas, Dimitrios Anagnostou, Magdalini Kreouzi, Sofia Kalantzi, Aikaterini Spyridaki, Maria Nikolaou

**Affiliations:** 1 Geriatric Outpatient Clinic 65+, Sismanogleio-Amalia Fleming General Hospital, Melissia, GRC

**Keywords:** access barrier, elderly patients, frailty syndrome, health care system, health inequalities, implementation challenges, social barrier

## Abstract

The aging population presents a growing challenge to healthcare systems, necessitating urgent adaptations to meet the complex needs of older adults. Existing healthcare models often lack integration and fail to provide patient-centered care, leading to fragmented services, suboptimal outcomes, increased hospitalizations, and escalating healthcare costs. This narrative review aims to systematically identify and categorize the key barriers to effective healthcare implementation for the elderly, evaluate current healthcare models and their limitations, and explore evidence-based strategies to improve care delivery. A comprehensive literature search was conducted in PubMed, MEDLINE, Scopus, and Web of Science for studies published from 2000 to October 2024. The identified barriers span multiple domains, including patient-related challenges such as low health literacy and socioeconomic disparities, disease-specific factors like frailty and multimorbidity, provider-related constraints such as inadequate geriatric training, and system-wide deficiencies in primary care infrastructure and policy support. To address these challenges, this review explores emerging solutions, including risk stratification tools, integrated healthcare models, digital health innovations, and artificial intelligence-driven interventions. By providing a structured analysis of barriers and solutions, this review aims to inform policy and healthcare practices that enhance elderly care, reduce hospital readmissions, and optimize resource utilization in aging populations.

## Introduction and background

The rapid aging of the global population presents a critical challenge to healthcare systems, necessitating urgent adaptations to meet the growing and complex needs of older adults. According to United Nations projections, by 2050, the number of people aged 65 and older will double to 1.55 billion, while the population aged 80 and above will triple to 426 million [1]. As a result, one in six people worldwide and one in four individuals in Europe and North America will be aged 65 or older [2].

Aging is associated with progressive physiological changes that increase susceptibility to adverse health outcomes. Older adults frequently experience multimorbidity, frailty, cognitive decline, polypharmacy, sarcopenia, malnutrition, and functional impairment, which not only complicate healthcare management but also contribute to increased hospitalization rates and reduced quality of life [3]. Socioeconomic constraints, such as limited financial resources post-retirement, further restrict healthcare access, promote sedentary lifestyles, and widen health inequities.

However, healthcare systems often fail to adequately address these complex needs, resulting in fragmented and inefficient care delivery. Existing models are frequently uncoordinated, leading to suboptimal health outcomes, higher hospitalization rates, and increased strain on healthcare infrastructure. This review aims to provide a structured analysis of the key barriers that hinder the effective implementation of healthcare for the elderly. Specifically, it examines challenges across multiple domains, including patient-related factors such as frailty and cognitive decline, healthcare system inefficiencies, provider limitations, and policy constraints. Furthermore, it explores current healthcare models and highlights evidence-based strategies, such as integrated care frameworks, digital health innovations, and risk stratification tools, that can enhance the delivery of care for older adults. By systematically categorizing these barriers and assessing potential solutions, this review seeks to inform policy development and healthcare practices that improve outcomes, reduce hospital readmissions, and optimize resource utilization in aging populations.

## Review

Methods

This narrative literature review examined barriers to effective healthcare implementation for elderly populations, focusing on frailty, cognitive impairment, multimorbidity, polypharmacy, care fragmentation, and technological accessibility. A comprehensive search was conducted in PubMed, MEDLINE, Scopus, and Web of Science, targeting peer-reviewed studies published from 2000 to October 2024. The search strategy incorporated both Medical Subject Headings (MeSH) terms for PubMed and free-text terms for all databases to ensure the comprehensive coverage of relevant topics and account for variations in indexing.

The search terms included key concepts related to elderly healthcare challenges, such as "elderly", "aging population", "geriatric care", and "healthcare challenges". Terms related to clinical conditions affecting elderly health included "frailty", "sarcopenia", "malnutrition", "multimorbidity", "non-communicable diseases", "polypharmacy", "functional decline", "cognitive impairment", "falls", and "non-adherence". Healthcare system-related issues were explored using terms such as "healthcare burden of aging", "fragmentation of care", "post-hospital syndrome", "transitional care", "hospital readmission", and "continuity of care". Interventions and care models were investigated using search terms like "integrated healthcare models", "patient-centered care", "medication reconciliation", and "multidisciplinary care teams". Lastly, technological and digital health solutions were covered using terms such as "telemedicine", "digital health technologies", "remote monitoring", "machine learning", and "artificial intelligence in elderly care".

Boolean operators (AND, OR) were employed to refine and expand search results appropriately, ensuring the retrieval of relevant literature. MeSH terms were applied where applicable, such as “frailty” [MeSH] OR “sarcopenia” [MeSH]. Studies were included if they examined barriers to effective healthcare for elderly populations or provided evidence-based strategies to improve care delivery. Reviews, meta-analyses, and primary research articles written in English with full-text availability were prioritized. Studies focusing exclusively on non-geriatric populations or lacking methodological transparency were excluded. To enhance rigor, three independent authors conducted the search and data extraction, resolving discrepancies through consensus. This approach ensured a broad yet targeted review of the literature, identifying key barriers to elderly care and evaluating strategies to mitigate these challenges (Table 1).

**Table 1:** Keywords and MeSH terms. MeSH: Medical Subject Headings

Category	Keywords	MeSH terms
Elderly healthcare challenges	elderly, aging population, geriatric care, healthcare challenges	Aged, Aging, Health Services for the Aged
Clinical conditions affecting elderly health	frailty, sarcopenia, malnutrition, multimorbidity, non-communicable diseases, polypharmacy, functional decline, cognitive impairment, falls, non-adherence	Frailty, Sarcopenia, Malnutrition, Multimorbidity, Cognitive Dysfunction, Polypharmacy
Healthcare system-related issues	healthcare burden of aging, fragmentation of care, post-hospital syndrome, transitional care, hospital readmission, continuity of care	Healthcare Disparities, Transitional Care, Continuity of Patient Care, Patient Readmission
Interventions and care models	integrated healthcare models, patient-centered care, medication reconciliation, multidisciplinary care teams	Comprehensive Health Care, Patient-Centered Care, Medication Reconciliation, Health Services Accessibility
Technological and digital health solutions	telemedicine, digital health technologies, remote monitoring, machine learning, artificial intelligence in elderly care	Telemedicine, Mobile Health, Remote Sensing Technology, Machine Learning, Artificial Intelligence

Characteristics of the vulnerable elderly population

Frailty

Frailty is a common syndrome among the elderly, characterized by progressive functional decline, decreased physiological reserve, and reduction in the resistance to endogenous and exogenous stressors, leading to an increase in an individual's vulnerability [3]. Approximately 11-15% of community-dwelling individuals over age 65 are affected by frailty [4]. The extra healthcare costs linked to frailty, separate from aging and coexisting health conditions, are estimated to be between €1,500 and €5,000 annually per person, depending on the care environment, underscoring the financial impact of managing frailty in elderly populations. Frailty may also bridge the gap between a person's chronological and biological age, making it essential to assess using established criteria rather than relying solely on clinical observation. Although no single, definitive method for evaluating frailty exists, numerous tools are available for clinical use. These tools vary in complexity, with some being thorough yet requiring considerable time and expertise and others being simpler but less detailed. The Fried phenotype, developed from the Cardiovascular Health Study and the Women's Health and Aging Study, is one widely recognized method, diagnosing frailty based on the presence of three or more out of five criteria: unintentional weight loss, fatigue, weakened grip strength, slowed walking speed, and low activity levels [3]. Pre-frailty is indicated when one or two of these criteria are met. Another method for assessing frailty considers the accumulation of various deficits, such as chronic conditions, cognitive impairment, malnutrition, and psychosocial factors, that contribute to a patient's overall vulnerability. In this model, the higher the number of accumulated deficits, the greater the level of frailty and the risk for adverse events like falls, hospitalization, and even death. Tools like Rockwood's Frailty Index provide a structured way to measure this cumulative effect [5,6].

Sarcopenia

In addition to frailty, sarcopenia is a notable condition with a prevalence of 6-12% in older adults [7]. Sarcopenia, an age-related decline in skeletal muscle mass, strength, and function, plays a crucial role in elderly health, with profound implications for mobility, independence, and overall quality of life. It is a multifactorial condition driven by a complex interplay of biological processes, including chronic inflammation, oxidative stress, hormonal dysregulation, neuromuscular degradation, and mitochondrial dysfunction. Notably, "inflammaging", a state of chronic low-grade inflammation observed in aging, contributes to muscle catabolism through the activation of nuclear factor kappa B (NF-κB) and the ubiquitin-proteasome pathway. Concurrently, hormonal changes, such as declines in growth hormone, testosterone, and insulin-like growth factor-1 (IGF-1), impair muscle protein synthesis, further accelerating sarcopenia [7]. Additionally, disuse atrophy due to physical inactivity, hospitalization, or bed rest exacerbates muscle loss, highlighting the importance of structured resistance training in prevention and management. Emerging evidence also underscores the impact of sarcopenia on metabolic health, as reduced muscle mass is associated with insulin resistance, increased adiposity, and higher cardiometabolic risk. Diagnostic challenges persist due to varying criteria across consensus groups, but key measures such as grip strength, gait speed, and dual-energy X-ray absorptiometry (DXA) assessments remain the gold standard. Interventions targeting sarcopenia include tailored exercise programs, adequate protein intake with a focus on leucine-rich sources, and pharmacological approaches such as myostatin inhibitors and anabolic agents under investigation. Given its significant contribution to frailty, falls, and mortality, sarcopenia should be recognized as a critical public health concern requiring early identification and comprehensive management strategies [7].

Malnutrition

Nutritional status plays a vital role in the health of older adults, significantly affecting their physical and cognitive functions, immune system, and overall quality of life. Malnutrition is a common concern in this population (3-30%) and can negatively impact outcomes, especially around surgical procedures. Various factors contribute to malnutrition among the elderly, including dental problems, difficulty swallowing, gastrointestinal changes, chronic illnesses, medications, psychological and cognitive challenges, and socioeconomic limitations. Comprehensive nutritional evaluation is an essential element of the Comprehensive Geriatric Assessment (CGA). The Mini Nutritional Assessment (MNA) is one of the most frequently used tools to evaluate nutritional health in older adults. It considers aspects such as recent weight loss, appetite, mobility, psychological stress, neuropsychological conditions, and body mass index (BMI), as well as dietary habits like protein and fluid intake [8]. Another common tool, the Malnutrition Universal Screening Tool (MUST), assesses nutritional risk based on BMI, unintentional weight loss, and the presence of acute illness that may lead to decreased food intake. Identifying malnutrition through these assessments allows healthcare providers to offer targeted nutritional support and physical training, particularly valuable in pre- and post-operative care for elderly patients [8,9]. 

Multimorbidity and Non-communicable Diseases (NCDs)

Multimorbidity, defined as the co-occurrence of two or more chronic conditions, is a common issue among elderly individuals, with its prevalence increasing significantly with age. According to a systematic review, in the majority of studies, over 50% of people aged 65+ are characterized by multimorbidity, reaching over 80-90% in some studies [10,11]. These comorbidities mostly include NCD, such as cardiovascular diseases, respiratory diseases, renal diseases, neurological and mental disorders, and musculoskeletal diseases. Multimorbidity poses complex challenges, as the interactions between various diseases and their treatments can lead to polypharmacy and potential drug-drug interactions, exacerbating health complications. Patients with multimorbidity often experience an increased risk of hospitalization by 10-20% per extra NCD, increased mortality by over 30-40% especially in the presence of multiple NCDs, as well as a decrease in functional status and quality of life [10,11].

Functional Decline

Functional status is a crucial predictor of health outcomes in elderly patients, indicating their ability to perform essential daily activities. Evaluating functional status provides clinicians with a baseline of a patient's physical capabilities and helps identify individuals at risk for complications after surgery, extended recovery periods, or potential loss of independence. Due to its importance, assessing functional status is a core part of the CGA [12]. The Barthel Index is a widely recognized tool for assessing basic activities of daily living (ADLs), covering 10 areas: feeding, bathing, grooming, dressing, bowel and bladder control, toilet use, transfers, mobility, and stair climbing. Another commonly used tool, the Katz Index, evaluates six basic ADLs, focusing on bathing, dressing, toileting, transferring, continence, and feeding. For more complex activities necessary for independent living, the Lawton Instrumental Activities of Daily Living (IADL) Scale is used. It assesses functions like using the phone, shopping, food preparation, housekeeping, laundry, transportation, managing medications, and handling finances. The Lawton Scale is particularly helpful for detecting subtle declines in function that might not be apparent through basic ADL assessments [12]. 

Cognitive Decline and Mental Health Disorders

Cognitive dysfunction is prevalent among older adults (5.1-41%), encompassing a spectrum of disorders from mild cognitive impairment to dementia. Cognitive decline can profoundly impact an individual's independence, mood, social interactions, sleep patterns, and overall quality of life. Advanced dementia is associated with additional complications, such as swallowing difficulties, malnutrition, increased aspiration risk, immobility, pressure sores, higher hospitalization rates, and mortality [13]. The CGA includes cognitive screening tools like the Mini Mental State Examination (MMSE), the Montreal Cognitive Assessment (MoCA), and the clock drawing test. The MMSE, widely used in cognitive screening, evaluates domains such as orientation, attention, calculation, recall, language, and command-following, with a score range from 0 to 30 and a commonly used threshold of 24 [14]. The MoCA offers a broader cognitive assessment, covering executive function, visuospatial skills, and attention, making it particularly effective for detecting early cognitive changes. The clock drawing test is another simple tool for identifying mild cognitive impairment; the patient is asked to draw a clock with a specific time, and scoring is based on accuracy [14]. 

Mental health disorders, including major depressive disorder and generalized anxiety disorder, are also common in elderly populations, significantly affecting quality of life, increasing substance abuse risk, and reducing medication adherence [15]. Assessing mental health is essential in the CGA, often performed using questionnaires and supplemented by a formal evaluation by a mental health professional. The Geriatric Depression Scale is specifically designed for identifying depressive symptoms in older adults, featuring a straightforward 30-question "Yes/No" format that makes it easy to use [16].

Polypharmacy and Medication Issues

Polypharmacy, the use of five or more medications, is a notable challenge in multimorbid elderly patients, associated with adverse effects and drug-drug interactions. Eurostat data show that prescribed medications are used by 87.1% and 46% of the populations aged 75+ and 45-54 years, respectively [17]. Furthermore, according to a systematic review, in the majority of studies, over 30-40% of people aged 65+ were characterized by polypharmacy, reaching over 50-60% in some studies [18]. However, despite the use of many medications, elderly and frail patients receive guideline-directed treatments at lower rates compared to younger and robust individuals. Specifically, a study from Dutch outpatients revealed that for every 10-year increase in age after 60, there was a 10-40% lower risk of receiving guideline-directed medical therapy (GDMT) for heart failure (HF) [19]. The above was confirmed in a Danish nationwide study, which showed similar results, together with the fact that age 80+ years was associated with a 30-40% higher risk of GDMT discontinuation [20]. The above-decreased rates of adherence to GDMT in the elderly are of particular interest. A recently published study from a Taiwan registry demonstrated that patients who received three or more HF-related medications had 50-60% reduced risks of one- and two-year readmission and 10-40% reduced risks of one- and two-year mortality irrespective of frailty status [21]. This highlights that elderly patients are marked by high rates of medication misuse, which involves overusing unnecessary drugs while simultaneously underusing guideline-directed treatments that are proven to improve their prognosis.

At this point, we should note that the elderly are at an increased risk of developing treatment complications, including drug side effects and post-interventional complications. A recent systematic review has demonstrated that elderly patients with cognitive impairment develop adverse drug reactions in up to 37% [22]. Furthermore, elderly and especially frail patients are at a higher risk of developing serious adverse effects from antihypertensive medications or medications used for the management of HF, including a 32% increased risk for hypotension, 20% increased risk for syncope, 44% increased risk for acute kidney injury, and 45% increased risk for electrolyte abnormalities [23,24]. 

As a result, we can summarize that elderly patients are in need of frequent medication reconciliation, to stop potentially dangerous and unneeded medications based on the Screening Tool of Older Persons' Prescriptions (STOPP)/Screening Tool to Alert to Right Treatment (START) and Beers criteria and prescribe necessary guideline-directed medications that can improve their prognosis. However, due to the risk of adverse effects, any commencement of new medications should be cautious, with the introduction of one drug at a time and frequent follow-up for adverse reactions. 

Underrepresentation in Clinical Trials

Another important phenomenon is that older adults are underrepresented in clinical trials. Data from 114 phase II and III trials showed that only 43.1% and 16.1% of the participants were aged 65+ and 75+ years, respectively. As a result, an increase in the representation of elderly and frail patients in future research should be a main goal of public health to avoid inequities and ensure the development of evidence-based practice guidelines for this demographic [25].

Falls

Falls are a significant issue for older adults, with around one-third of people aged 65 and older experiencing at least one fall each year and 10% experiencing multiple falls annually. The risk is particularly high in individuals in their 80s and 90s, where the annual fall incidence can reach 50%. Of these falls, approximately 30-50% result in minor injuries, while 10-20% lead to more severe injuries, such as fractures or head trauma. Hip fractures occur in about 1% of all falls among the elderly, which significantly increases the risk of post-fall morbidity and mortality. Risk factors for falls in the elderly population include a history of previous falls, muscle weakness, impaired balance, cognitive impairment, and environmental hazards such as poor lighting or slippery surfaces. Age-related changes in vision, proprioception, and reflexes, combined with chronic conditions such as Parkinson's disease, osteoarthritis, postural hypotension, and cardiovascular disorders, further exacerbate the risk of falls. Polypharmacy, especially the use of sedatives, antidepressants, and antihypertensives, also contributes significantly to fall risk by causing dizziness, orthostatic hypotension, and confusion. Post-fall syndrome, characterized by fear of falling and subsequent reduced mobility, can lead to a decline in physical activity and functional independence, creating a vicious cycle [26]. Finally, unexplained falls should undergo the same diagnostic work-up as syncope according to the 2018 European Society of Cardiology (ESC) guidelines for syncope, focusing on the exclusion of severe underlying cardiac structural or electrophysiological abnormalities [27].

Non-adherence

The elderly often achieve suboptimal adherence mostly due to the following several barriers: (1) Cognitive impairment can hinder a patient's ability to process and retain information, leading to errors in taking medications [13]. (2) Sensory deficits such as hearing loss or poor vision can make it difficult for patients to fully comprehend verbal or written instructions [13]. (3) Polypharmacy, the use of five or more medications, usually associated with complex treatment regimens, is common among the elderly introducing further adherence issues. Medication side effects or interactions, combined with a lack of understanding of the need for each medication, can further discourage adherence [28]. (4) Socioeconomic factors such as low income, limited access to healthcare services, and poor social support can make it difficult for elderly patients to fill prescriptions, attend follow-up appointments, or engage in necessary lifestyle changes. Lack of transportation, physical limitations, and reliance on caregivers for assistance also contribute to poor adherence in elderly populations [29]. 

Healthcare burden of the increasing aging population

Based on Eurostat data, from 2001 to 2019, the average annual number of hospitalizations across all age groups in Europe declined by 13.2%, from 2,964,866.4 to 2,574,480.1. This reduction likely reflects advancements in healthcare, including improved preventive measures, better outpatient management, and guideline-directed therapy for prevalent diseases.

However, this trend varied significantly across age groups. While hospitalizations decreased by 8.5%, 15.4%, and 12.6% in individuals aged 60-69, 70-74, and 75-79 years, respectively, a notable increase was observed in older age groups. Specifically, hospitalization rates rose by 22.3% in individuals aged 80-84, 20% in those aged 85-89, 26% in those aged 90-94, and a striking 58.7% in those aged 95 and older. Collectively, the number of hospitalizations among individuals aged 80+ years increased by 23.2%, corresponding to an additional 95,168 hospitalizations over this period.

**Figure 1:**
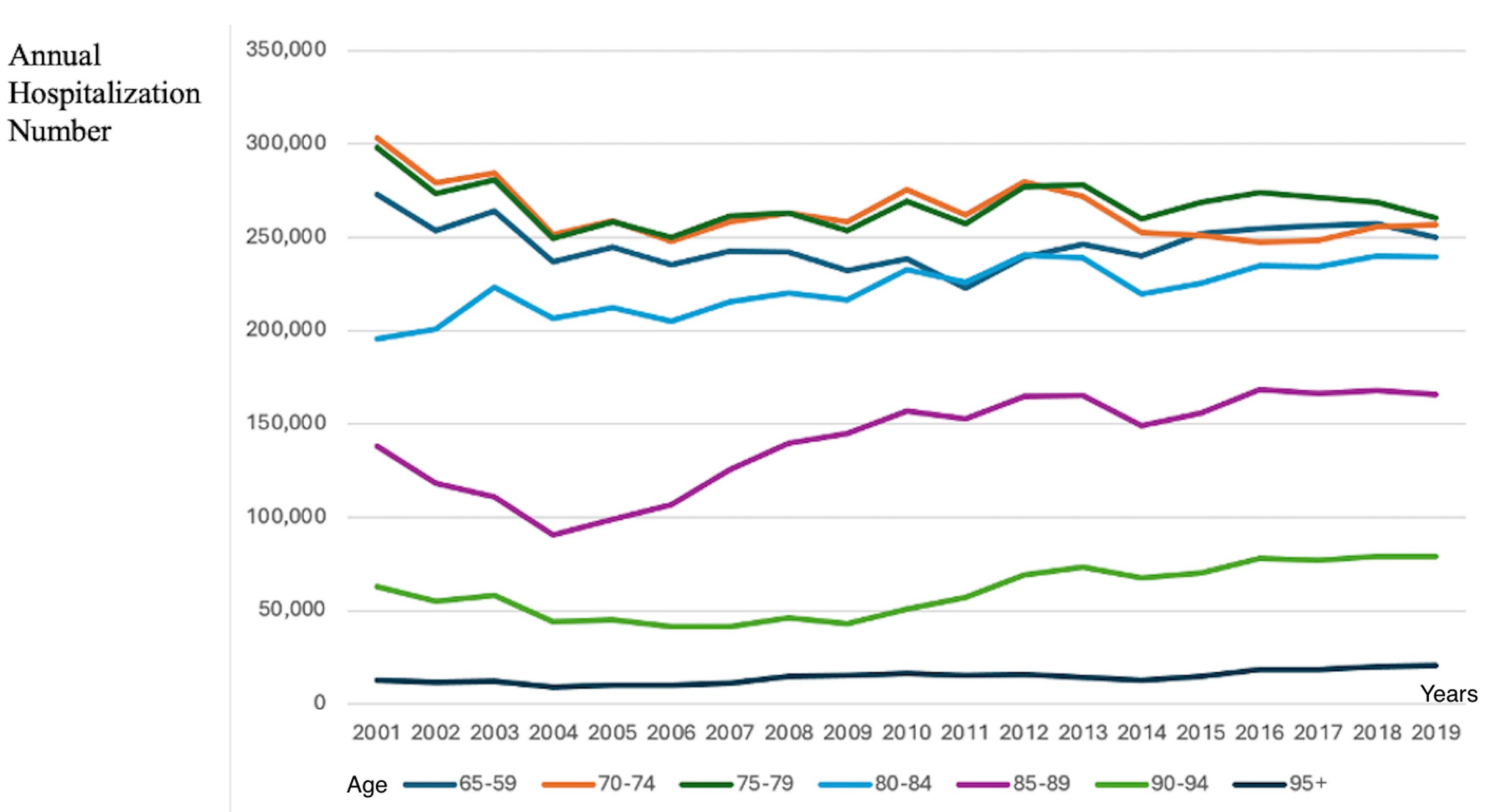
Trends in the average annual number of hospitalizations in Europe from 2001 to 2019. This figure illustrates the annual number of hospitalizations in Europe from 2001 to 2019, stratified by age group. The data show a declining trend in hospitalization rates among younger elderly populations (65-79 years), while hospitalizations among older age groups (80+ years) exhibit a progressive increase over time. Notably, individuals aged 90-94 and 95+ years demonstrate the most pronounced rise in hospitalization numbers, reflecting the increasing healthcare burden associated with aging. These trends highlight the growing demand for geriatric care and the need for integrated healthcare models to manage multimorbidity and frailty in older adults.

These data highlight a shifting healthcare burden toward the very elderly population, emphasizing the growing demand for specialized geriatric care, integrated healthcare models, and hospital resource optimization to accommodate the increasing complexity and healthcare needs of aging individuals. The trends in hospitalization rates across age groups from 2001 to 2019 are summarized in Table 2 and illustrated in Figure 1 [17].

**Table 2:** Trends in the average annual number of hospitalizations in Europe from 2001 to 2019. This table presents the mean number of annual hospitalizations across various age groups in Europe between 2001 and 2019. The data illustrate an overall 13.2% decline in total hospitalizations, with significant reductions observed in younger elderly populations (aged 65-79 years). In contrast, hospitalization rates have increased substantially in individuals aged 80+ years, particularly in those 90 years and older, where the trend shows a 58.7% increase.

Mean European number of hospitalizations	2001	2019	Trend
Total	2,964,866.4	2,574,480.1	-13.2%
65-59	273,086.1	249,917.6	-8.5%
70-74	303,435.9	256,777.5	-15.4%
75-79	298,132.6	260,469.5	-12.6%
80-84	195,729.6	239,349.6	22.3%
85-89	138,157.9	165,836	20%
90-94	62,695.1	79,004.5	26%
95+	12,872.8	20,434.2	58.7%

**Figure 2:**
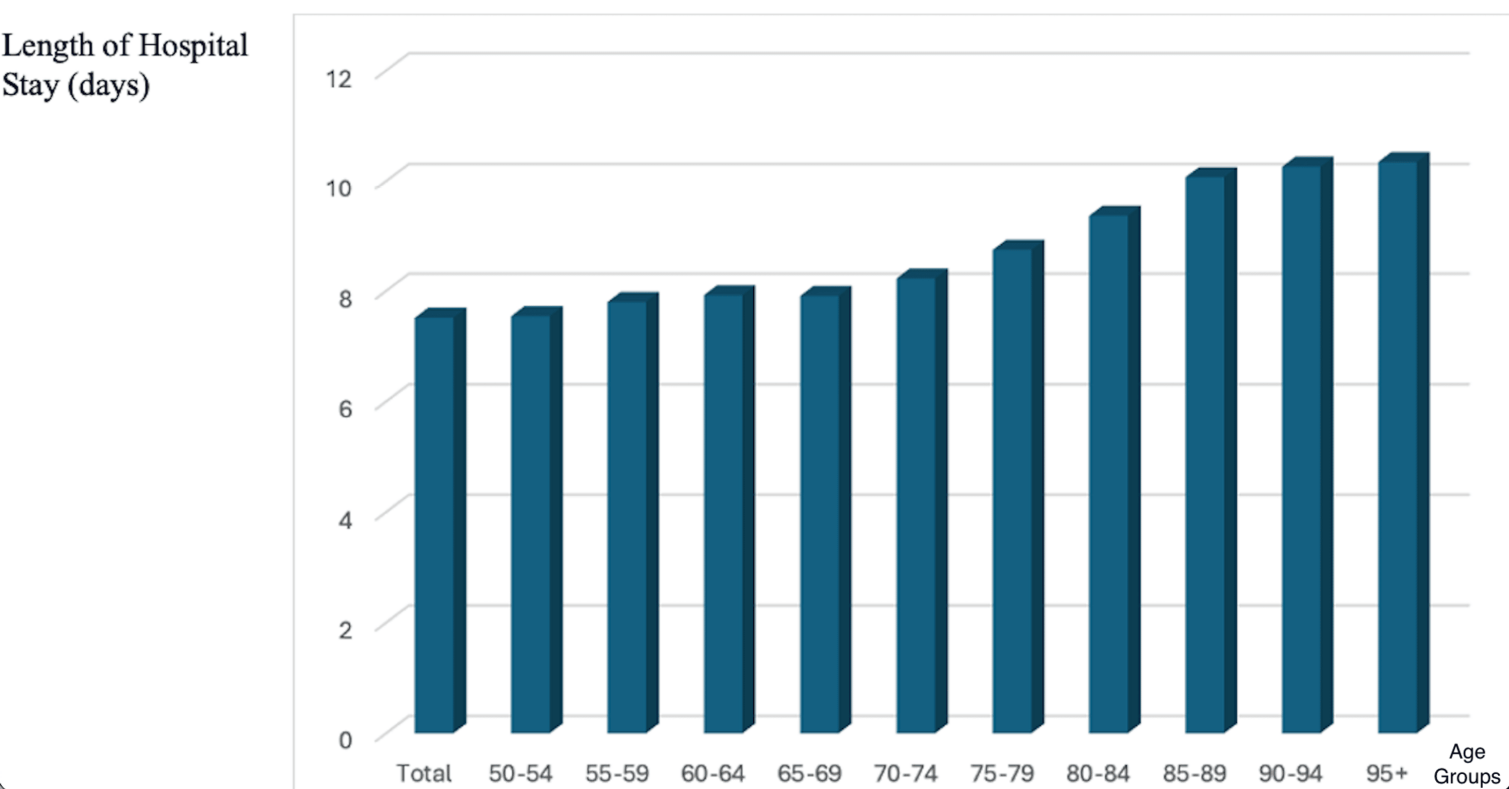
Average hospital length of stay (days) by age group for 2021 in Europe. This figure presents the average duration of hospitalizations across different age groups in Europe for 2021. The data show a progressive increase in hospital length of stay with advancing age, with patients aged 50-64 years having relatively stable hospitalization durations, while those aged 75 years and older experience a marked rise. The longest hospital stays are observed in individuals aged 90+ years, reflecting the impact of frailty, multimorbidity, and increased healthcare complexity in the elderly population. These findings highlight the need for targeted inpatient care strategies and enhanced post-discharge planning to optimize recovery and reduce hospital burden in older adults.

According to Eurostat data for 2021, the average hospital length of stay increases progressively with age. Patients aged 50-54 years had a mean hospital stay of 7.55 days, while those aged 80 years and older had an average stay of approximately 10 days. The data reveal a steady rise in hospitalization duration across older age groups, with the longest lengths of stay observed in individuals aged 90-94 and 95+ years. This trend reflects the increasing complexity of care, multimorbidity, frailty, and functional decline associated with advanced age, necessitating longer inpatient management and rehabilitation. The findings underscore the growing demand for specialized geriatric care, hospital resource allocation, and post-hospitalization strategies to improve outcomes in the elderly. The relationship between age and hospital length of stay is illustrated in Figure 2 [17]. 

A notable trend in hospitalization rates is the sharp increase with advancing age. In 2021, the average annual hospitalization rate in Europe was approximately 10% among individuals aged 50-54, but it rose dramatically to over 50% in those aged 85 and older. This means that one in two individuals aged 85+ is hospitalized every year, reflecting the increasing healthcare burden associated with aging.

**Figure 3:**
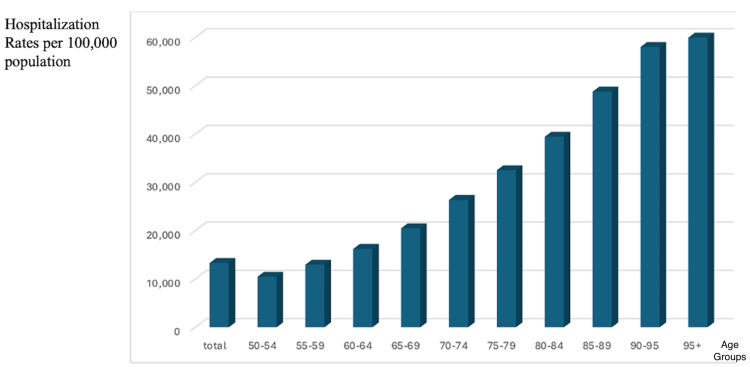
Mean age-specific annual hospitalization rates per 100,000 population for 2021 in Europe. This figure illustrates the age-related increase in hospitalization rates across Europe in 2021. The data show that hospitalization rates remain relatively low in middle-aged adults (50-64 years) but rise progressively in older age groups. By age 85+, over 50% of individuals experience at least one hospital admission annually, highlighting the significant healthcare burden associated with aging. These findings emphasize the need for proactive geriatric care strategies, early intervention, and post-discharge support to reduce hospital dependency and improve health outcomes in the elderly.

These findings support the rising mean age of hospitalized patients, emphasizing a more complex inpatient profile with higher rates of multimorbidity, frailty, and functional decline. Additionally, the increased length of hospital stay and healthcare costs per admission further illustrate the growing demand for specialized inpatient management, rehabilitation services, and efficient hospital resource allocation to accommodate the needs of the aging population. The relationship between age and hospitalization rates is depicted in Figure 3.

According to Eurostat data for the European Union (27 countries), in 2019, hospital care expenditure amounted to €351.1 billion, representing 25.2% of total healthcare expenditure, or €785.43 per inhabitant. Considering forecasting models that predict the population aged 80 and older is expected to triple by 2050, reaching 426 million, the expected annual global expenditure for hospital care could potentially increase by €111.13 billion compared to 2019 [17]. 

The above data highlights the need for future health policies to focus on managing the healthcare needs of the continuously growing aging population. 

Fragmentation of care

The elderly, multimorbid, frail patients are the most frequent users of healthcare systems, requiring outpatient and in-hospital coordinated health services from several healthcare providers. Consequently, they constitute the group that suffer the most from the fragmentation of care, defined as care that is diffusely spread across many physicians, such that no single physician accounts for a substantial proportion of visits [30]. To manage their diversity of chronic conditions, elderly patients require the attention of different specialists, and their care might also involve informal caregivers, and third-sector carers, unavoidably leading to care fragmentation. This care is frequently not coordinated, with each specialist focusing narrowly on their area of expertise without adequately considering the overall health and care plan of the patient. This can lead to conflicting medical advice, polypharmacy, drug-drug interactions, and unnecessary duplication of diagnostic tests or interventions, ultimately resulting in suboptimal care and worse patient outcomes and inevitably to a rise in health costs [30,31]. Additionally, there is a distinction between health and social care services, both of which are essential for the elderly. Yet, these sectors frequently operate independently of each other, further contributing to care fragmentation and missed opportunities for integrated care. A recently published Danish nationwide cohort demonstrated that clinical indicators of care fragmentation (e.g., number of contacts, involved providers, transitions, and hospital trajectories) increased with the number of comorbidities. Furthermore, this was the first study to show that fragmentation of care is associated with increased mortality and inappropriate medication use, after adjusting for confounders. Additionally, the everyday routine of an elderly frail person usually must encompass the contribution of a physiotherapist, a 24-hour caregiver, and a psychologist with experience in mental empowerment services that are not covered by health insurances, raise the cost of living, but enhance substantially the quality of life of this population [31]. 

In addition to care fragmentation in the outpatient sector, the transition from primary care to hospital settings and vice versa is often marked by poor continuity of care. Primary care physicians (PCPs) play a critical role in the long-term management of chronic diseases and in monitoring the overall health of elderly patients. However, during hospital admissions, particularly in acute settings, the PCPs' insights into the patient's baseline condition, medication regimen, and long-term management plans may not always be effectively communicated to hospital teams. Similarly, following hospital discharge, elderly patients frequently experience a breakdown in communication between hospital specialists and their PCPs. Hospital discharge letters and changes in medications are often poorly communicated with PCPs, while follow-up care plans are often delayed or incomplete. This can result in medication errors, drug adverse effects, drug-drug interactions, missed follow-up appointments, and, ultimately, increased rates of rehospitalization [32,33]. We should also note that sometimes the follow-up of these patients presupposes their transportation to hospital facilities which is an extra uncovered cost for patients with mobility problems, thus making this very difficult and expensive.

Post-hospital syndrome (PHS) and readmissions

PHS, first reported by Harlan M. Krumholz in 2013, is a transient, generalized condition of vulnerability experienced by patients following hospital discharge that is associated with an increased risk of adverse health outcomes, including readmissions, within 30 days of discharge. For elderly patients, particularly those with multiple NCDs or frailty, PHS can lead to a cycle of recurrent hospitalizations, reduced functional status, and poor overall prognosis [34]. 

PHS arises from the stressors experienced during hospitalization, which include the acute condition itself as well as disruptions in sleep patterns, inadequate nutrition, dehydration, social isolation, pain, delirium, physical deconditioning due to immobilization, medication changes, interactions and side effects, and the psychological stress of the process of hospitalization. These factors collectively impair the recovery process, especially for frail patients which are characterized by reduced reserve, resulting in the development of infections, increased risk of falls, and exacerbations of chronic diseases in the weeks following discharge [34]. 

According to Medicare data, the risk of 30-day readmission is approximately 20% in the general population, with higher rates at elderly and frail patients [35,36]. The top causes for 30-day readmission rates include HF, chronic obstructive pulmonary disease (COPD) exacerbation, pneumonia, and myocardial infarction. Interestingly, the 15% of 30-day readmissions seem potentially avoidable [37,38]. 

Noteworthy, the cause of readmission is usually different than the cause of index hospitalization. According to Medicare data, for patients with an index hospitalization for HF, pneumonia, COPD, or gastrointestinal disorders, the cause of 30-day readmission is a different condition in 63%, 70.9%, 63.8%, and 78.9% of cases, respectively [34,35]. Among all causes of index hospitalization collectively, HF demonstrated the highest rate of 30-day readmission (26.9%). Furthermore, HF was the most common cause of all readmissions collectively (8.6%). 

According to a recent retrospective study, common risk factors for readmission include a higher Charlson Comorbidity Index (12% increased risk by additional point), polypharmacy (66% higher risk), living in the community with home care (61% increased risk), length of stay was five days or longer (72% increased risk), being discharged on a Friday (88% increased risk), or being discharged from a surgical unit (2.09-fold risk) [39]. Another cohort demonstrated that pre-hospital factors rather than hospital factors account for the increased risk of 30-day readmission. These factors include acute index admission, recent hospital admission (the higher the risk, the lower the days since discharge), male gender, low personal income, unemployment, higher Charlson Comorbidity Index, higher number of prescribed drugs, higher number of previous 30-day readmissions, and higher number of PCP visits or calls [40]. 

**Figure 4:**
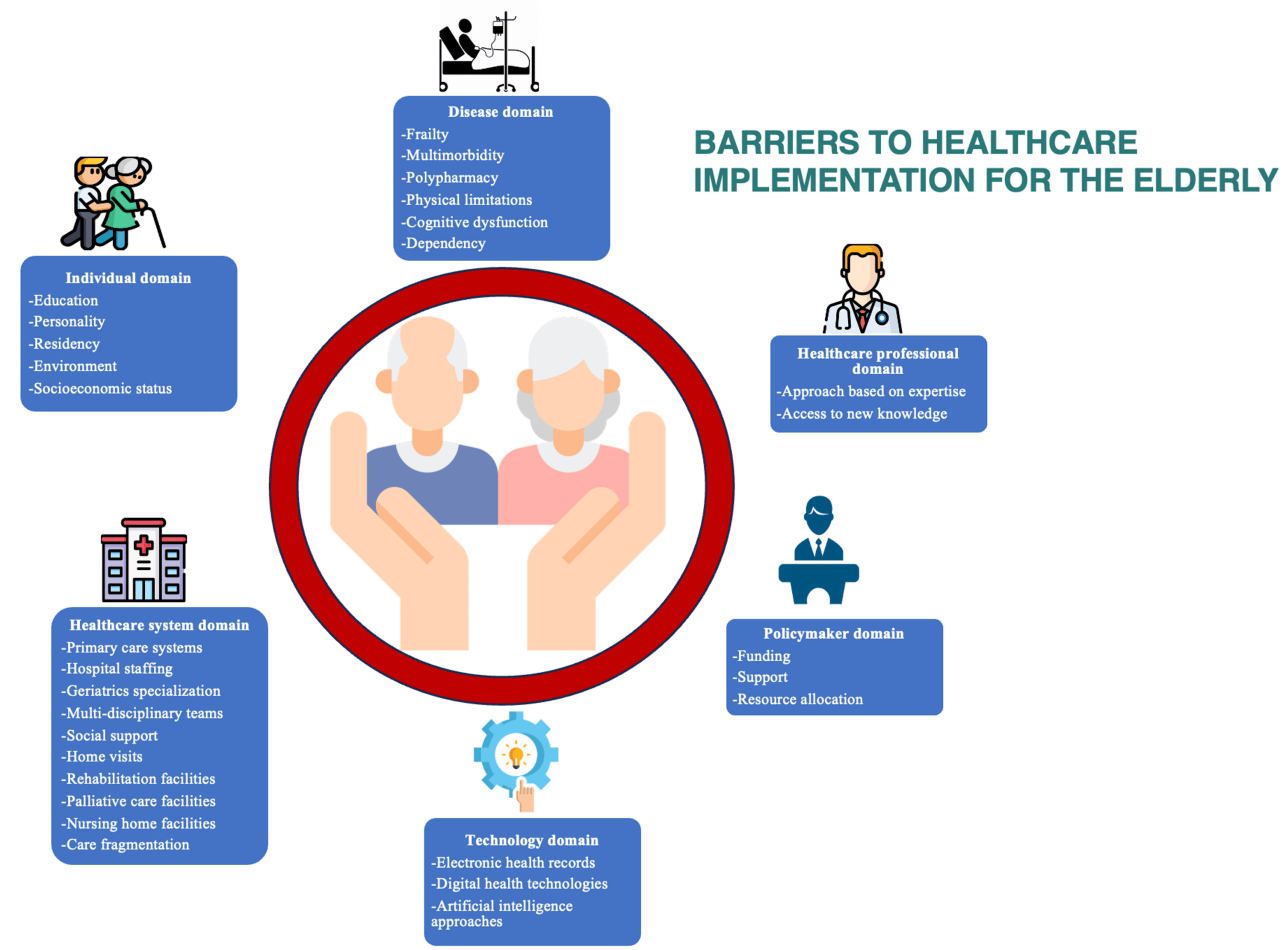
Key domains of barriers to an effective implementation of healthcare in the elderly population. Source: Author's own creation.

The key domains of barriers to an effective implementation of healthcare in the elderly are presented in Table 3 and illustrated in Figure 4. 

**Table 3:** Key domains of barriers to an effective implementation of healthcare in the elderly population. References: [6-64]

Domain	Barriers
Individual domain	Educational level
	Personality
	Place of residence (e.g., remote areas)
	Type of residence (e.g., home, nursing home)
	Presence of a supportive environment/caregivers
	Socioeconomic status: limited financial resources can restrict access to healthcare resources, digital health tools, and follow-up care
Disease domain	Frailty
	Multimorbidity
	Polypharmacy
	Physical limitations: mobility issues can limit access to in-person follow-up visits, especially if transportation is a barrier
	Cognitive dysfunction: memory impairments and cognitive decline in elderly patients can hinder their ability to absorb and retain information
	Level of dependency
	Post-hospitalization syndrome
Healthcare professional domain	Approach based on specialization
	Access to up-to-date guidelines and knowledge
Healthcare system domain	Presence of structured primary healthcare
	Availability of geriatric specialization and geriatric centers
	Availability of a diversity in healthcare professionals both in the hospital and in the community (physicians, community nurses, clinical pharmacists, physiotherapists, clinical nutritionists, occupational therapists, speech therapists, social workers, psychologists)
	Presence of multidisciplinary teams
	Presence of a structured social support system
	Presence of a structured program for home visits (including community nurses)
	Presence of rehabilitation facilities
	Presence of facilities for palliative care
	Presence of structured nursing home facilities
	Sufficient staffing of hospitals
	Fragmented or integrated care
Technology domain	Availability of electronic health records
	Availability of digital health technologies, including mHealth, mobile patient monitoring, and artificial intelligence approaches
Financial, institutional, and policymaker domain	Availability of funding for elderly care and research adequacy of policy support for elderly care
	Resource allocation constraints

Key strategies to improve healthcare for the elderly

Since elderly patients are frequently hospitalized, using risk stratification tools to identify patients at high risk for hospitalizations can allow for targeted interventions. Specifically, elderly patients with multiple NCDs, frailty, or cognitive impairment should receive extra support during and after hospitalization [41]. Additionally, comprehensive discharge planning is crucial and should ensure that patients are discharged with clear, comprehensive plans for follow-up care, medication management, and physical rehabilitation. According to a recent meta-analysis, communication interventions at discharge were significantly associated with 31% lower readmission rates, 24% higher adherence to treatment regimens, and 41% higher patient satisfaction [42]. 

Furthermore, implementing transitional care programs that provide coordinated follow-up after discharge can help ensure continuity of care and improve outcomes. These programs often involve home visits by healthcare professionals, telemedicine follow-up, and patient education on managing chronic conditions and medications. A recent meta-analysis demonstrated that continuity of care interventions significantly reduced rehospitalizations by 16% at one month and by 26% at three months [43]. According to a recent cohort, having a post-hospitalization PCP visit at 14 and seven days was associated with a significantly lower hospital 30-day readmission risk by 32% and 24%, respectively, and a significantly lower hospital 90-day readmission risk by 25% and 20%, respectively [44]. Furthermore, another study demonstrated that post-hospitalization follow-up 20-minute visits by PCPs within seven days are significantly associated with a 28% lower risk for 30-day readmission [45]. Notably, an additional cohort demonstrated that attending at least one post-hospitalization PCP visit significantly decreased the odds of readmission by 50%, while attending at least one specialist visit decreased the odds of readmission by 100% [46]. This highlights the need of both primary and specialized post-hospitalization care, ideally in the context of a multidisciplinary team (MDT). 

Integrated care is an umbrella term implying an attempt to coordinate and integrate fragmented and piecemeal health systems with new organizational arrangements, including primary care, hospitals, social services, and specialty care [47]. Coordination of care involves an approach in which physicians are collectively operating in a team-like manner to develop and implement an overall care plan to meet the patient's goals. Some strategies include better communication protocols, the use of digital health technologies and shared electronic health records (EHRs), and care models that are based on MDT [48]. 

Ensuring accurate medication reconciliation can prevent errors, reduce adverse drug reactions, and ensure patients are adherent to their prescribed regimens. A recent meta-analysis demonstrated that the risk of medication error in patients who underwent medication reconciliation was 75% lower than those receiving usual care [49]. 

An MDT involves collaboration among professionals from various healthcare and social domains to deliver holistic patient care. MDT approaches can be classified as multidisciplinary (professionals work independently within their specialties), interdisciplinary (collaborative, interactive, and goal-oriented teamwork), or transdisciplinary (integrated roles beyond specific disciplines). For older adults with multiple NCDs, MDT approaches are crucial to address complex health needs and improve quality of life. In the 2024 study by Hayes et al., MDT integrated care with general practitioner (GP) participation showed notable benefits: over 12-36 months, functional status improved by 21% (standardized mean difference (SMD): 0.21), hospitalizations decreased by 23% (risk ratio (RR): 0.77), and patient satisfaction significantly rose (SMD: 0.46). These findings highlight MDT effectiveness in domains like physical health, care coordination, and patient-centered support, emphasizing the value of coordinated community-based interventions for older populations [50]. 

Patient education and engagement are foundational to modern healthcare, especially for elderly patients managing multiple NCDs. Educating patients about their conditions, treatment regimens, potential side effects, and the importance of adhering to therapies can drastically improve health outcomes and enhance overall quality of life. With aging populations, healthcare providers increasingly face patients with complex health profiles who need continuous support to manage chronic conditions effectively [51]. By fostering an understanding of their health, patient education enables individuals to take a proactive role in their care, transforming them from passive recipients of medical advice to active participants in managing their own health. This empowerment is particularly important in modern medicine, where a more collaborative patient-provider relationship is recognized as essential to successful treatment outcomes [44]. Educated patients are often more engaged, asking informed questions and communicating openly about symptoms or concerns. They are also better equipped to make informed decisions, helping avoid potential complications that may arise from misunderstandings about their health status or therapies. Patient engagement further promotes adherence to treatment plans and encourages lifestyle modifications, like diet or exercise, that can prevent disease progression [51,52]. Moreover, this involvement in their own care can improve psychological well-being, as patients often experience a greater sense of control and motivation when they are empowered with knowledge. In today's healthcare landscape, patient education not only supports the individuals in managing their NCDs effectively but also can potentially relieve strain on healthcare systems by reducing unnecessary emergency department visits. Patient education and engagement can be effectively supported through a variety of tools and methodologies that make health information accessible, interactive, and actionable for elderly patients. These methods can include educational brochures and videos, patient portals, digital apps, telemedicine and virtual health coaching, wearable devices, educational workshops and support groups, and teach-back methods [51,52]. 

Improving adherence can have a significant impact on prognosis. A meta-analysis has demonstrated that medication adherence interventions among patients with HF can significantly reduce mortality risk by 11% and the risk of readmission by 21%. Improving patient education and adherence requires a multifaceted approach [53]. Some effective strategies include the following: (1) Simplified communication by using clear, straightforward language and providing written materials that are easy to read can help patients better understand their treatment plans. Visual aids, such as diagrams, videos, or infographics, can also be highly effective in communicating complex health information, especially in patients with hearing loss [54]. (2) Medication management tools such as pill organizers, medication calendars, and automated reminders (e.g., smartphone apps or calls) can help patients adhere to complicated medication schedules. Regular medicine reconciliation can also help reduce the burden of polypharmacy and minimize the risk of drug interactions or adverse effects [54]. (3) Including caregivers in the education process ensures that they are equipped to help the patient with medication adherence, lifestyle changes, and appointment scheduling [54]. (4) Providing assistance for transportation to appointments, offering telehealth options, connecting patients with social services or community support programs, and providing home health visits by physicians or community nurses can address some of the socioeconomic factors that contribute to poor adherence [54]. (5) Continuous follow-up and regular monitoring are essential to ensuring adherence. Scheduled check-ins via phone/telehealth or the use of mobile health (mHealth) can help reinforce the importance of sticking to prescribed therapies and address any new concerns that may arise. A recent meta-analysis has demonstrated that mHealth interventions in HF patients can significantly improve medication adherence by 26% and reduce the risk of readmission by 37% [55]. 

Novel technologies

The integration of novel technologies into elderly healthcare has the potential to significantly enhance well-being, promote independent living, and improve health outcomes. However, adoption remains limited due to several barriers, including poor user interface design, security concerns, affordability, and digital literacy challenges. Many elderly individuals may lack experience with digital tools, struggle with complex navigation, or have concerns regarding data privacy, which hinder them from fully utilizing these technologies. Additionally, financial constraints may limit access to smart devices, internet services, and subscription-based health platforms, particularly in low-resource settings.

Digital health encompasses a broad range of technologies, including telemedicine, mHealth, EHRs, wearable devices, and remote monitoring systems [56]. 

Telemedicine enables healthcare providers to deliver care remotely, addressing barriers such as transportation difficulties and geographic isolation, which are common among elderly patients. Video consultations, mobile apps, and telephone follow-ups can help older adults manage chronic conditions and maintain continuity of care post-hospitalization. mHealth applications provide self-management tools, medication reminders, and real-time health monitoring, empowering elderly patients to adhere to treatment regimens and engage actively in their own care [56]. However, adoption can be limited by low digital literacy and usability challenges. To enhance accessibility, healthcare systems can implement simplified interfaces, hands-on digital literacy training, and community-based tech support programs.

Wearable devices that track vital signs (e.g., heart rate, blood pressure, oxygen saturation) and physical activity levels are invaluable for monitoring elderly patients with NCDs. Remote monitoring systems can alert healthcare providers to early signs of deterioration, such as hypotension, tachycardia, or desaturation, allowing for timely interventions. Additionally, fall-detection devices provide immediate alerts to caregivers or healthcare professionals in case of accidents, improving safety and response times [57]. Ensuring affordability and ease of use through government subsidies or insurance coverage can help expand access to these beneficial technologies.

EHRs and patient portals enhance care coordination for elderly patients, particularly those with multimorbidity, by consolidating their medical history, medication lists, and treatment plans in one place. These technologies reduce the risk of care fragmentation and medication errors, allowing multiple providers to access up-to-date patient information. Additionally, patient portals enable elderly individuals and caregivers to schedule appointments, access medical records, and communicate with healthcare providers [58]. To facilitate adoption, user-friendly interfaces with larger fonts, voice-assisted navigation, and simplified functionalities can improve engagement and usability for older adults.

Machine learning (ML) and artificial intelligence (AI) have the potential to enhance clinical decision-making, predict adverse outcomes, and tailor interventions to individual patient needs. Some applications include the following: (1) Hybrid ML models integrating variables such as medical history, laboratory examinations, and social determinants of health can accurately detect specific profiles of elderly patients who have the highest risk of hospitalization and rehospitalization. This enables healthcare providers to target these high-risk patients with early interventions, such as enhanced post-discharge care or transitional care programs, potentially reducing readmission rates [59]. (2) ML models can be used to analyze medication data and provide a list of drug-drug interactions [60]. (3) ML algorithms can aid in customizing rehabilitation programs, monitoring progress, and predicting individual patient needs [61]. ML models can analyze data from cognitive and physical assessments or even voice patterns to detect early signs of cognitive decline [62]. (4) Combining AI with remote monitoring tools can further improve the management of elderly patients. For instance, AI-powered devices can continuously analyze data from wearables or home sensors to detect subtle changes in a patient's health status, such as alterations in sleep patterns, mobility, or heart rate variability. These applications can help detect patients with worsening functional status or patients with an acute condition that needs medical attention [63]. 

To enhance the adoption of digital health solutions among the elderly, several strategies should be implemented such as (1) Improving digital literacy through training programs, community workshops, and personalized tech support to help older adults navigate healthcare technologies, (2) enhancing affordability by providing subsidized devices, insurance coverage, or publicly funded telehealth programs to expand access, (3) simplifying technology interfaces with user-friendly designs, voice-assisted features, and large-text displays to accommodate the needs of elderly users, and (4) ensuring data privacy and security by implementing strong protection policies, transparent data-sharing practices, and patient education on cybersecurity to alleviate concerns.

The COVID-19 pandemic accelerated digital literacy among elderly individuals, creating a unique opportunity to integrate digital health technologies into routine care. As older adults have adapted to virtual platforms, they are now better positioned to benefit from telehealth, remote monitoring, and mHealth applications. By capitalizing on this shift and tailoring digital interventions to elderly populations, healthcare systems can improve accessibility, engagement, and health outcomes, ensuring that technology plays a central role in supporting aging populations worldwide [64].

A summary of the key strategies for improving healthcare for the elderly is summarized in Table 4.

**Table 4:** Key strategies for improving healthcare for the elderly MDT: multidisciplinary team; EHRs: electronic health records; AI: artificial intelligence

Strategy	Description	Impact
Risk stratification	Using risk assessment tools to identify elderly patients at high risk for hospitalization, allowing for targeted interventions	Reduces preventable hospitalizations and improves proactive care management
Comprehensive discharge planning	Ensuring patients receive clear, structured plans for follow-up care, medication management, and rehabilitation to reduce readmissions	Decreases readmission rates, enhances medication adherence, and improves patient satisfaction
Transitional care programs	Coordinating post-discharge follow-up through home visits, telemedicine, and patient education to improve care continuity	Improves long-term health outcomes by reducing rehospitalization rates and strengthening care coordination
Post-hospitalization primary care follow-up	Ensuring timely primary care visits within 7-14 days post-hospitalization to significantly lower the risk of readmission	Significantly reduces 30- and 90-day readmission risks by improving early post-discharge care
Integrated care models	Coordinating care across healthcare sectors (hospitals, primary care, social services) to prevent fragmentation and improve patient outcomes	Minimizes fragmented care, leading to better health outcomes and efficient healthcare resource utilization
Medication reconciliation	Reducing medication errors and adverse reactions by reviewing medications at transitions of care and discontinuing inappropriate prescriptions	Lowers the risk of adverse drug reactions and improves prescription accuracy in elderly patients
MDT approach	Engaging specialists, primary care providers, nurses, and allied health professionals in a structured, interdisciplinary care model	Enhances patient-centered care by incorporating multiple disciplines into decision-making and treatment plans
Patient education and engagement	Educating elderly patients about disease management, medication adherence, and lifestyle modifications to improve engagement and self-care	Empowers elderly patients to take an active role in their healthcare, leading to improved adherence and quality of life
Improving medication adherence	Implementing tools such as pill organizers, medication reminders, caregiver involvement, and mobile health interventions to improve adherence	Reduces the risk of treatment discontinuation, prevents complications, and improves long-term patient outcomes
Telemedicine and digital health	Using telehealth and mobile health apps for remote consultations, patient education, and continuous care monitoring	Increases access to healthcare, especially for elderly individuals in remote areas, improving continuity of care
Wearable and remote monitoring devices	Leveraging wearable devices to track vital signs, detect falls, and enable early interventions for deteriorating health conditions	Enables continuous monitoring, early detection of deterioration, and timely medical interventions
EHRs and patient portals	Enhancing care coordination through real-time access to medical history, treatment plans, and appointment scheduling via patient portals	Reduces duplication of medical tests, prevents medication errors, and fosters better doctor-patient communication
AI and machine learning	Utilizing AI-driven predictive analytics for early risk detection, medication optimization, and personalized rehabilitation plans	Optimizes treatment strategies, identifies at-risk patients earlier, and personalizes healthcare interventions

Generalizability

The vulnerabilities and barriers to healthcare implementation typically associated with aging are not exclusive to the elderly. Younger individuals with chronic and severe conditions, such as HF, disabling stroke, chronic kidney disease, COPD, advanced malignancies with cachexia, neurodegenerative disorders, and severe mental health conditions, often exhibit similar levels of frailty and health-associated risks. This overlap allows for the adaptation of geriatric care principles to these younger, similarly vulnerable populations, broadening the applicability of specialized care strategies beyond just older adults.

Additionally, the proposed strategies, including integrated care models, MDT-based approaches, and patient education, can be tailored to low-resource settings to enhance their feasibility and effectiveness. In such environments, leveraging community health workers, digital health interventions, and simplified screening tools can help bridge healthcare gaps, optimize resource allocation, and improve patient outcomes. Strengthening primary healthcare infrastructure, task-shifting strategies, and culturally adapted patient education programs can further enhance the global applicability of these interventions, ensuring that effective healthcare solutions extend to diverse populations regardless of healthcare system constraints. This broader approach underscores the need for scalable and adaptable strategies to improve healthcare access and outcomes across various settings.

## Conclusions

Addressing the healthcare needs of a rapidly aging population requires a shift toward more integrated, patient-centered care approaches that account for the unique vulnerabilities of elderly patients. The prevalence of frailty, multimorbidity, cognitive impairment, and polypharmacy among older adults highlights the need for coordinated healthcare delivery models that involve MDT and continuity of care across different settings. Technological innovations such as telemedicine, mHealth, and wearable health monitors offer promising solutions for enhancing patient engagement, adherence, and monitoring, yet accessibility and ease of use must be prioritized to ensure effectiveness among elderly users. Additionally, implementing patient education strategies and supportive resources can empower elderly patients to actively participate in managing their health, potentially reducing adverse outcomes and hospital readmissions.

Beyond addressing elderly care, adopting geriatric principles in healthcare can benefit younger patients facing similar vulnerabilities due to chronic and complex health conditions. However, it is crucial to recognize potential biases in existing research, including the underrepresentation of elderly and frail individuals in clinical trials, disparities in healthcare access across different socioeconomic groups, and the predominance of studies conducted in high-income countries. These factors may limit the generalizability of findings and emphasize the need for more inclusive research efforts that better reflect the diverse aging population. Moving forward, healthcare systems must adapt to the demands of an aging world by prioritizing evidence-based policies, adequate funding, and resource allocation to improve care for elderly populations while fostering inclusivity for all patients with similar care needs.
